# Effects of dietary protein restriction on muscle fiber characteristics and mTORC1 pathway in the skeletal muscle of growing-finishing pigs

**DOI:** 10.1186/s40104-016-0106-8

**Published:** 2016-08-22

**Authors:** Yinghui Li, Fengna Li, Li Wu, Hongkui Wei, Yingying Liu, Tiejun Li, Bie Tan, Xiangfeng Kong, Kang Yao, Shuai Chen, Fei Wu, Yehui Duan, Yulong Yin

**Affiliations:** 1 Key Laboratory of Agro-ecological Processes in Subtropical Region, Institute of Subtropical Agriculture, Chinese Academy of Sciences; Hunan Provincial Engineering Research Center for Healthy Livestock and Poultry Production; Scientific Observing and Experimental Station of Animal Nutrition and Feed Science in South-Central, Ministry of Agriculture, No. 644 Yuanda Road, Furong District, Changsha, Hunan 410125 China; 2 University of Chinese Academy of Sciences, Beijing, 100039 China; 3 Hunan Co-Innovation Center of Animal Production Safety, CICAPS; Hunan Collaborative Innovation Center for Utilization of Botanical Functional Ingredients, Changsha, 410128 China; 4 College of Animal Sciences, Huazhong Agricultural University, Wuhan, Hubei 430070 China; 5 School of Biology, Hunan Normal University, Changsha, Hunan 410018 China

**Keywords:** Dietary protein restriction, Energy status, Growing-finishing pigs, mTORC1, Muscle fiber type

## Abstract

**Background:**

To investigate the effects of dietary crude protein (CP) restriction on muscle fiber characteristics and key regulators related to protein deposition in skeletal muscle, a total of 18 growing-finishing pigs (62.30 ± 0.88 kg) were allotted to 3 groups and fed with the recommended adequate protein (AP, 16 % CP) diet, moderately restricted protein (MP, 13 % CP) diet and low protein (LP, 10 % CP) diet, respectively. The skeletal muscle of different locations in pigs, including *longissimus dorsi* muscle (LDM)*, psoas* major muscle (PMM) and *biceps femoris* muscle (BFM) were collected and analyzed.

**Results:**

Results showed that growing-finishing pigs fed the MP or AP diet improved (*P* < 0.01) the average daily gain and feed: gain ratio compared with those fed the LP diet, and the MP diet tended to increase (*P* = 0.09) the weight of LDM. Moreover, the ATP content and energy charge value were varied among muscle samples from different locations of pigs fed the reduced protein diets. We also observed that pigs fed the MP diet up-regulated (*P* < 0.05) muscular mRNA expression of all the selected key genes, except that myosin heavy chain (*MyHC) IIb*, *MyHC IIx*, while mRNA expression of ubiquitin ligases genes was not affected by dietary CP level. Additionally, the activation of mammalian target of rapamycin complex 1 (mTORC1) pathway was stimulated (*P* < 0.05) in skeletal muscle of the pigs fed the MP or AP diet compared with those fed the LP diet.

**Conclusion:**

The results suggest that the pigs fed the MP diet could catch up to the growth performance and the LDM weight of the pigs fed the AP diet, and the underlying mechanism may be partly due to the alteration in energy status, modulation of muscle fiber characteristics and mTORC1 activation as well as its downstream effectors in skeletal muscle of different locations in growing-finishing pigs.

**Electronic supplementary material:**

The online version of this article (doi:10.1186/s40104-016-0106-8) contains supplementary material, which is available to authorized users.

## Background

The skeletal muscle, which accounts for 20–50 % of total body mass, is the primary metabolic tissue, contributing up to 40 % of the resting metabolic rate [1, 2]. Meanwhile, it also acts as an endocrine organ, regulating the disposal of nutrient and energy consumption in the body by secreting myokines, such as interleukin (IL)-6 and IL-15 [3, 4]. Thus, development and maintenance of skeletal muscle are crucial for body health and life quality [5].

Mammalian target of rapamycin complex 1 (mTORC1) plays a key role in protein synthesis of skeletal muscle [6, 7], and constitutively consist of mTOR, regulatory associated protein of mTOR (Raptor), and mLST8/GβL [8, 9]. In brief, Raptor functions positively in mTORC1 pathway by acting as an adaptor to recruit substrates to mTOR [8, 9]. Additionally, mTORC1 promotes mRNA translation through multiple downstream effectors such as eukaryotic initiation factor (eIF) 4E-binding protein1 (4E-BP1) and p70S6 kinase (S6K1), resulting in protein synthesis [10]. On the other hand, protein degradation in skeletal muscle is primarily mediated by activation of the ubiquitin (Ub)-proteasome pathway (UPP). There are two specific E3 ubiquitin ligases belonging to the UPP, muscle ring finger 1 (MuRF1) and muscle atrophy F-box (MAFbx), both are proposed to be central to the control of muscle proteolysis [11]. Actually, protein deposition depends on the balance between the rates of protein synthesis and degradation.

It is well known that feeding-induced stimulation of protein deposition is most pronounced in skeletal muscle [12]. Several studies reported that a high protein diet contributed to muscle mass raise [13–18]. However, over the past few years, some studies pointed out that a high protein diet failed to translate into muscle mass [19, 20], suggesting that a high protein intake may not necessarily lead to accumulation of muscle protein. In addition, numerous studies showed that chronic feeding of a low protein diet impaired activation of translation initiation, consequently reducing protein synthesis [21]. Besides, maternal low protein diet during gestation and lactation could regulate myostatin pathway and protein synthesis in skeletal muscle of offspring at weaning stage [22]. All the findings confirmed that a very important role for dietary protein level in modulating protein metabolism and muscle growth, but less is known about the mechanisms of protein deposition and myofiber development of the pigs influenced by a moderately restricted protein diet.

In general, skeletal muscle fiber types are distinguished according to the predominantly expressed isoform of myosin heavy chain (MyHC), which are referred to as type I, IIa, IIx and IIb [23–25]. Myofiber type proportions of *longissimus dorsi* muscle (LDM)*, psoas* major muscle (PMM) and *biceps femoris* muscle (BFM) are varied due to their anatomical location (Additional file 1: Table S1) and thus they have different metabolic properties. Previous research conducted in the rats during periods of fasting observed that the degree of reduction in protein synthesis was not similar in various muscles [26, 27]. This led us to hypothesize that the expression levels for MyHC and muscle development regulators genes could specifically modulated in different muscle of pigs by protein-restricted diet. Therefore, the present study aimed to investigate the effects of restricted protein diet on growth performance, muscle fiber characteristics and protein expression of key molecules related to mTORC1 pathway in different locations of skeletal muscle of pigs.

## Methods

All experimental procedures in the present study were reviewed and approved by the Animal Care and Use Committee of the Chinese Academy of Sciences.

### Animals and diets

A total of 18 crossbred barrows (Large White × Landrace × Duroc, 62.30 ± 0.88 kg) were randomly divided into three treatments. Each treatment had six replicates (*n* = 6). Animals were housed individually in cages and fed isocaloric diets based on corn-soybean meal (Table 1). The dietary treatments were as follows: 1) a recommended adequate protein (AP) diet containing 16 % crude protein (CP); 2) a moderately restricted protein (MP) diet containing 13 % CP (3 % unit reduction); 3) a low protein (LP) diet containing 10 % CP (6 % unit reduction). In addition, the limiting amino acids including lysine, methionine, threonine and tryptophan were supplemented to meet the requirements of National Research Council (NRC 2012). The ratios of essential amino acids to non-essential amino acids are 0.70, 0.74 and 0.78, respectively. All the growing-finishing pigs had *ad libitum* access to diet and water throughout the study. All pigs were weighed at the start and the end of the 50-day experiment, and feed intake was recorded every 2 wk to calculate average daily gain (ADG), average daily feed intake (ADFI), and the Feed:Gain ratio (F:G).

**Table 1:** Ingredient composition and nutrient levels of the experimental diets (as-fed basis, %)

Items	Protein levels^a^		
	AP (16 % CP)	MP (13 % CP)	LP (10 % CP)
Ingredient composition, %			
Corn	67.00	78.36	87.40
Soybean meal	23.76	15.00	5.50
Wheat bran	6.00	3.00	2.00
Soybean oil	0.88	0.90	1.71
Lys	0.01	0.27	0.55
Met	0.00	0.00	0.09
Thr	0.00	0.06	0.19
Trp	0.00	0.01	0.06
CaHPO_4_	0.50	0.55	0.65
Limestone	0.55	0.55	0.55
NaCl	0.30	0.30	0.30
1%Premix^b^	1.00	1.00	1.00
Total	100.00	100.00	100.00
Nutrient levels, %			
DE(MJ/kg)^c^	14.20	14.20	14.20
CP	16.30	13.17	10.26
Lys	0.72	0.72	0.73
Met + Cys	0.50	0.42	0.43
Thr	0.56	0.50	0.49
Trp	0.17	0.13	0.13
Arg	0.94	0.70	0.44
His	0.39	0.31	0.22
Ile	0.60	0.45	0.30
Leu	1.32	1.13	0.91
Phe	0.71	0.57	0.41
Val	0.61	0.50	0.36
Total calcium	0.52	0.50	0.51
Total phosphorus	0.45	0.40	0.38
Essential AA	6.29	5.24	4.23
Non-essential AA	8.76	7.10	5.36
Essential/Non-essential AA	0.70	0.74	0.78

^a^AP = adequate protein; MP = moderately restricted protein; LP = low protein

^b^Supplied per kg of diet: CuSO_4_ · 5H_2_O 19.8 mg; KI 0.20 mg; FeSO_4_ · 7H_2_O 400 mg; NaSeO_3_ 0.56 mg; ZnSO_4_ · 7H_2_O 359 mg; MnSO_4_ · H_2_O 10.2 mg; Vitamin K (menadione) 5 mg; Vitamin B_1_ 2 mg; Vitamin B_2_ 15 mg; Vitamin B_12_ 30μg; Vitamin A 5,400 IU; Vitamin D_3_ 110 IU; Vitamin E 18 IU; Choline chloride 80 mg; Antioxidants 20 mg; Fungicide 100 mg

^c^Caculated values

### Tissue sample collection

At the end of the trial, all the pigs were fasted overnight and sacrificed. Pigs were stunned by electrical shock (250 V, 0.5 A, for 5 to 6 s), exsanguinated, and eviscerated in a slaughterhouse. The head was removed, and the carcass was split longitudinally. Skeletal muscle tissue including LDM*,* PMM and BFM were dissected and weighted. Samples (about 2 × 1 × 1 cm) from LDM, PMM, and BFM were rapidly excised and immediately frozen in liquid nitrogen, then stored at -80 °C until analysis.

### Measurement of ATP, ADP and AMP levels of skeletal muscle

Contents of ATP, ADP and AMP in skeletal muscle were determined according to previous publications with some modification [28]. Tissue extracts (100 mg) were prepared from frozen LDM, PMM and BFM (within 1 wk after slaughter), using 1 mL 5 % perchloric acid, and the extracts were centrifuged at 13,000 × g for 8 min at 4 °C. The supernatants were neutralized with 2 mol/L KHCO_3_ and centrifuged again. The standards of ATP (FLAAS), ADP (A5285), AMP (01930) were purchased from Sigma (Sigma-Aldrich, MO, USA). High performance liquid chromatography (HPLC) was performed with a reverse-phase column (99603, C18, 5 μm, 250 × 4.6 mm, Dikma Technologies Inc.) and the column temperature was set at 25 °C. For measurements of metabolites, a mobile phase consisting of 215 mmol/L KH_2_PO_4_, 1.2 mmol/L tetrabutylammonium bisulfate, 1 % acetonitrile (pH 6.0) was used and the flow rate was maintained at 0.8 mL/min by a HPLC pump (600E; Waters, MA, USA). Eluted samples were detected at 260 nm with a dual λ absorbance detector (2478, Waters). Calibration curves were prepared by a six-point standard (0.2, 0.1, 0.05, 0.025, 0.0125 and 0.00625 mg/mL) of ATP, ADP and AMP in 0.6 mol/L perchloric acid, respectively. Total energy charge (EC) was calculated according to the equation: EC = (ATP + 0.5ADP)/(ATP + ADP + AMP).

### RNA extraction and cDNA synthesis

Total RNA was isolated from LDM, PMM, and BFM samples using TRizol Reagent (Invitrogen-Life Technologies, CA, USA) [29, 30]. The integrity of RNA was evaluated by 1 % agarose gel electrophoresis. The concentration of the extracted RNA was checked by spectrophotometry using NanoDrop ND2000 (NanoDrop Technologies Inc., DE, USA), and purity of RNA was assessed by using the A260/A280 ratio, which ranged from 1.8–2.0. About 1.0 μg of total RNA was incubated with DNase I (Fermentas, WI, USA), and later used for reverse transcription using First-Strand cDNA Synthesis Kit according to the manufacturer’s protocol. The cDNA was synthesized with Oligo dT and superscript II reverse-transcriptase, and the cDNA were stored at -80 °C before further processing.

### Quantitative real-time PCR

The relative mRNA expression levels of target genes were analyzed by quantitative real-time PCR with specific primers designed using Premier 5.0 (Table 2). Real-time quantitative PCR was done using with ABI 7900HT Real-Time PCR system (Applied Biosystems, Branchburg, NJ, USA), and performed using SYBR Green detection kit (Thermo, MA, USA) according to the manufacturer’s instructions. The reaction program was as follows: incubation for 10 min at 95 °C, followed by 40 cycles of denaturation for 15 s at 95 °C, annealing and extension for 60 s at (56–64 °C). The reaction mixture lacking cDNA was used as a negative control in each run. Each sample was measured in duplicate. The gene expression level was calculated using the comparative (2^-ΔΔCT^) method [31]. The house-keeping gene *β-actin* was used as internal control.

**Table 2:** The Primers used for real-time PCR analysis

Genes	Primer sequences (5’→3’)	Product size, bp	Accession NO.
*MyHC I*	F: GGCCCCTTCCAGCTTGA	63	L10129
	R: TGGCTGCGCCTTGGTTT		
*MyHC IIa*	F: TTAAAAAGCTCCAAGAACTGTTTCA	100	U11772
	R: CCATTTCCTGGTCGGAACTC		
*MyHC IIx*	F: AGCTTCAAGTTCTGCCCCACT	76	U90719
	R: GGCTGCGGGTTATTGATGG		
*MyHC IIb*	F:CACTTTAAGTAGTTGTCTGCCTTGAG	80	U90720
	R: GGCAGCAGGGCACTAGATGT		
*MyoD*	F: CAACAGCGGACGACTTCTATG	383	NM001002824
	R: GCGCAAGATTTCCACCTT		
*MyoG*	F: GCAGGGTGCTCCTCTTCA	230	NM001012406
	R: AGGCTACGAGCGGACTGA		
*IL-6*	R: AGTTGAAGGTGGTCTCGTGG	118	NM001252429
	R: TCTGCCAGTACCTCCTTGCT		
*IL-15*	F: GCATCCAGTGCTACTTGTGT	118	NM214390
	R: TGCCAGGTTGCTTCTGTTTT		
*MuRF1*	F:AGCACGAAGACGAGAAAATC	150	NM001184756
	R:TGCGGTTACTCAGCTCAGTC		
*MAFbx*	F:CCAGAGAGTCGGCAAGT	374	NM001044588
	R:GAGGGTAGCATCGCACAAGT		
*β-actin*	F: TGCGGGACATCAAGGAGAAG	216	XM003357928.2
	R: AGTTGAAGGTGGTCTCGTGG		

*MyHC* myosin heavy chain, *MyoD* myoblast determination protein, *MyoG* myogenin, *IL-6* interleukin-6, *IL-15* interleukin-15, *MuRF1* muscle ring finger 1, *MAFbx* muscle atrophy F-box

### Western blotting analysis

Western blot analysis was conducted according to the previous study [4]. Briefly, about 30–50 μg of total protein extracted from muscle samples was separated by reducing SDS-PAGE electrophoresis. Western blots were incubated with primary antibodies rabbit anti- phospho (p)-mTOR (Ser2448, Cell Signaling Technology, USA), p-Raptor (Ser863, Santa Cruz Biotechnology, USA), p-4E-BP1 (Ser65, Cell Signaling Technology, USA) or p-S6K1 (Thr389, Cell Signaling Technology, USA) at a dilution of 1:1,000 after blocking with 5 % nonfat milk. The membranes were then rinsed in TBST and incubated with second antibody peroxidase-conjugated anti-goat or anti-rabbit IgG (Santa Cruz Biotechnology, Santa Cruz, CA) for 1 h at a dilution of 1:5,000. For examining the equal loading, mouse anti-β-actin (Santa Cruz Biotechnology, USA) diluted at 1:5,000 was used as internal control. Finally, the bands of the protein were visualized using a chemiluminescent reagent (Pierce, Rockford, USA) with a ChemiDoc XRS system (Bio-Rad, Philadelphia, PA, USA). We quantified the resultant signals using Alpha Imager 2200 software (Alpha Innotech Corporation, CA, USA) and normalized the data with inner control.

### Statistical analysis

Data obtained from this study was analyzed by the One-way analysis of variance (ANOVA) using SAS 8.2 software (Cary, NC, USA) followed by a Duncan’s multiple comparison test. Differences between significant means were considered as statistically different at *P* < 0.05 and a trend toward significance at *P* < 0.10.

## Results

### Growth performance and skeletal muscle mass weight

The growth performance and the weight of the skeletal muscle mass are presented in Table 3. Final body weight and ADFI of pigs fed the LP diet was lower (*P* < 0.05) than those fed the AP diet, but there were no significant differences of those two parameters between the MP and AP groups. The ADG was decreased (*P* < 0.01) with the dietary protein level reduction, and improved (*P* < 0.01) F:G was observed in pigs fed the MP or AP diet compared with those fed the LP diet. In terms of the skeletal muscle mass weight, PMM and BFM did not show any significant differences among the treatments, whereas a trend to significance (*P* = 0.09) was noted in LDM of pigs fed the MP diet compared with those fed the AP or LP diet.

**Table 3:** Growth performance and skeletal muscle mass weight of growing-finishing pigs fed the restricted protein diets^1^

Items	Dietary treatment^2^			SEM^3^	*P* value
	AP (16 % CP)	MP (13 % CP)	LP (10 % CP)		
Growth Performance					
Initial body weight, kg	62.33	62.30	62.28	0.88	0.99
Final body weight, kg	101.43^a^	97.88^ab^	94.02^b^	1.45	<0.01
Average daily gain, g/d	782.00^a^	711.67^b^	634.33^c^	16.84	<0.01
Average daily feed intake, g/d	2, 819.30^a^	2, 614.70^ab^	2, 547.50^b^	76.50	0.05
Feed:Gain ratio	3.60^b^	3.67^b^	4.02^a^	0.06	<0.01
Muscle Weight					
*Longissimus dorsi* muscle, g	2854.3^b^	3182.13^a^	2922.5^b^	66.57	0.09
*Psoas* major muscle, g	437.03	455.33	404.37	11.35	0.18
*Biceps femoris* muscle, g	1683.9	1621	1582.37	28.16	0.35

^1^Means within different superscript letters are significantly different at *P* < 0.05 and a trend toward significance at *P* < 0.10, *n* = 6

^2^AP = adequate protein; MP = moderately restricted protein; LP = low protein

^3^SEM = standard error of mean

### Contents of ATP, ADP, AMP and EC value in skeletal muscle

As shown in Table 4, significant changes in ATP content and EC value were observed among LDM, PMM and BFM. Notably, the pigs fed the MP diet markedly raised (*P* < 0.01) the ATP content and EC value in LDM compared with those fed the LP or AP diet. As for PMM, the AP diet-fed pigs significantly elevated (*P* < 0.01) the ATP content and EC value. However, for BFM, the AP or MP groups markedly decreased (*P* < 0.01) the ATP content compared with that of the LP group, and the EC value of the AP group was higher (*P* < 0.05) than that of the LP group. The contents of ADP and AMP in the skeletal muscle of different locations were not affected by dietary CP level, except that the AMP content in LDM was increased (*P* < 0.05) in pigs when dietary protein level reduced.

**Table 4:** Effect of the restricted protein diets on the content of ATP, ADP, AMP and EC value in the skeletal muscle of different locations of growing-finishing pigs^1^

Parameter, ppm	Dietary treatment^2^			SEM^3^	*P* value
	AP (16 % CP)	MP (13 % CP)	LP (10 % CP)		
*Longissimus dorsi* muscle					
ATP	49.56^b^	172.35^a^	85.19^b^	13.41	<0.01
ADP	312.26	314.65	243.31	24.41	0.10
AMP	50.30^b^	65.08^ab^	87.97^a^	8.57	0.02
EC	0.50^b^	0.60^a^	0.50^b^	0.01	<0.01
*Psoas* major muscle					
ATP	106.54^a^	59.06^b^	75.23^ab^	14.47	0.07
ADP	189.75	181.14	181.28	16.83	0.92
AMP	89.57	97.42	113.81	10.24	0.26
EC	0.52^a^	0.44^b^	0.45^b^	0.01	<0.01
*Biceps femoris* muscle					
ATP	33.17^b^	42.45^b^	67.66^a^	5.81	<0.01
ADP	262.07	289.66	280.42	14.77	0.43
AMP	56.31	57.56	65.84	5.93	0.48
EC^4^	0.47^b^	0.48^ab^	0.50^a^	0.01	0.02

^1^Means within different superscript letters are significantly different (*P* < 0.05). *n* = 6

^2^AP = adequate protein; MP = moderately restricted protein; LP = low protein

^3^SEM = standard error of mean

^4^EC = energy charge. EC was calculated using the following formula: EC = (ATP + 0.5ADP)/(ATP + ADP + AMP)

### Gene mRNA expression of MyHC isoforms

**Fig. 1:**
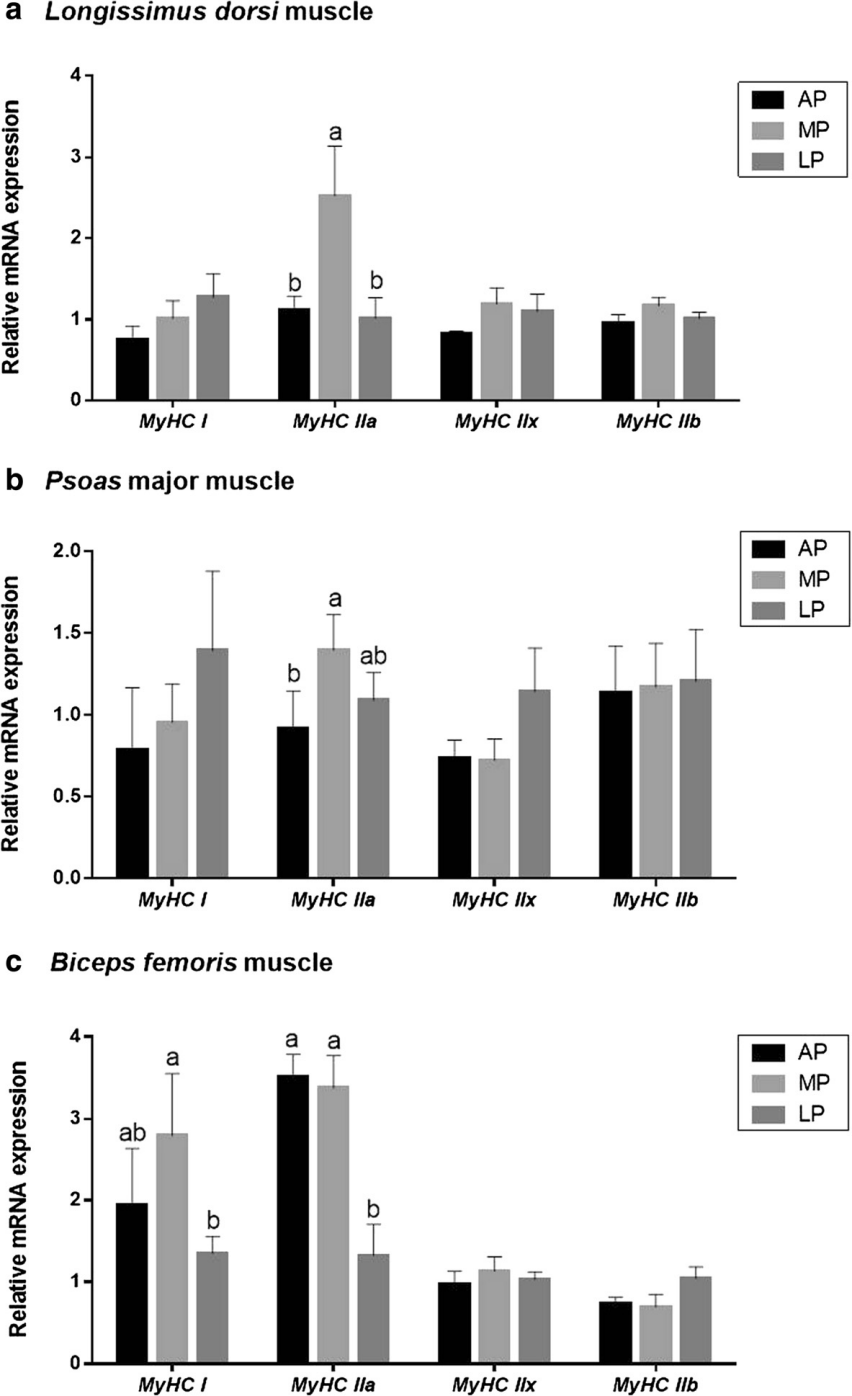
Relative mRNA expression of myosin heavy chain isoform (*MyHC I*, *IIa*, *IIx*, and *IIb*) in the skeletal muscle of different locations of growing-finishing pigs fed the restricted protein diets. *β-actin* was used as an internal control. Data are expressed as means ± SE (*n* = 6). Bars with different letters are significantly different at *P* < 0.05. MyHC = myosin heavy chain; AP = adequate protein; MP = moderately restricted protein; LP = low protein

As shown in Fig. 1, the mRNA expression of oxidative fiber isoforms (*MyHC I* and *IIa*) were significantly regulated by the dietary CP level in LDM, PMM and BFM of the pigs. For the mRNA expression of* MyHC I*, it was increased (*P* < 0.05) in BFM of pigs fed the MP diet compared with those fed the LP diet, but there was no difference between the MP and AP groups. For the value of *MyHC IIa*, it was higher (*P* < 0.05) in LDM of pigs fed the MP diet compared with those fed the AP or LP diet; the value was also higher (*P* < 0.05) in PMM of MP-fed pigs, but no difference was observed between the MP and LP-fed pigs; the value was increased (*P* < 0.05) in BFM of pigs fed the AP or MP diet compared with those fed the LP diet. However, there were no differences in the mRNA levels of *MyHC IIx* and *IIb* among the treatments in the skeletal muscle of different locations.

### Myokines, myogenic regulatory factors (MRFs) and E3 ubiquitin ligases associated genes mRNA expression

**Fig. 2:**
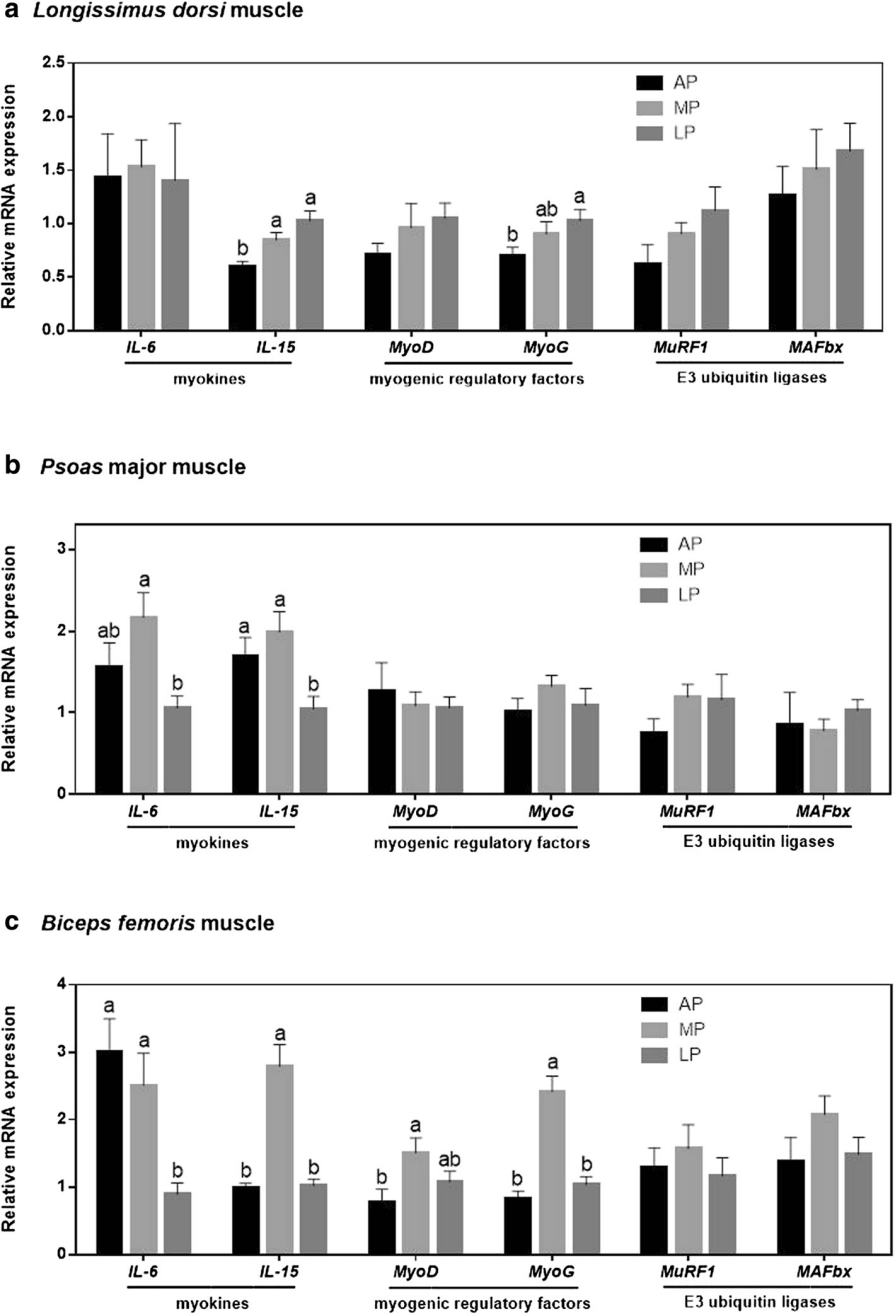
Relative mRNA expression of genes coding for myokines (*IL-6*, *IL-15*), myogenic regulatory factors (*MyoD*, *MyoG*), and ubiquitin ligases (*MuRF1*, *MAFbx*) in the skeletal muscle of different locations of growing-finishing pigs fed the restricted protein diets. *β-actin* was used as an internal standard to normalize the signal control. Data are expressed as means ± SE (*n* = 6). Bars with different letters are significantly different at *P* < 0.05. IL-6 = interleukin-6; IL-15 = interleukin-15; MyoD = myoblast determination protein; MyoG = myogenin; MuRF1 = muscle ring finger 1; MAFbx = muscle atrophy F-box; AP = adequate protein; MP = moderately restricted protein; LP = low protein

In LDM, the mRNA level of *IL-15* was significantly decreased (*P* < 0.05) in AP-fed pigs compared with LP or MP-fed pigs, and the value of *MyoG* was also lower (*P* < 0.05) in AP-fed pigs than that in LP-fed pigs, but no difference was noted between LP and MP groups (Fig. 2a). In PMM, the mRNA expression level of *IL-6* was higher (*P* < 0.05) in pigs fed the MP diet compared with those fed the LP diet, but there was no difference between those fed the AP and MP diet; the value of *IL-15* was decreased (*P* < 0.05) in LP-fed pigs relative to that of AP or MP-fed pigs (Fig. 2b). In BFM, the mRNA level of *IL-6* was raised (*P* < 0.05) in AP or MP-fed pigs compared with that of LP-fed pigs; while the values of* IL-15* and *MyoG* were the highest (*P* < 0.05) in pigs fed the MP diet compared with those fed the AP or LP diet; the mRNA level of *MyoD* in AP-fed pigs was decreased (*P* < 0.05) relative to MP-fed pigs, but there was no difference between MP and LP groups (Fig. 2c). Additionally, restricted protein diets did not influence the mRNA expression of E3 ligases (*MuRF1* and *MAFbx*) in the skeletal muscle of different locations in growing-finishing pigs.

### Protein expression of key molecules related to mTORC1 pathway

**Fig. 3:**
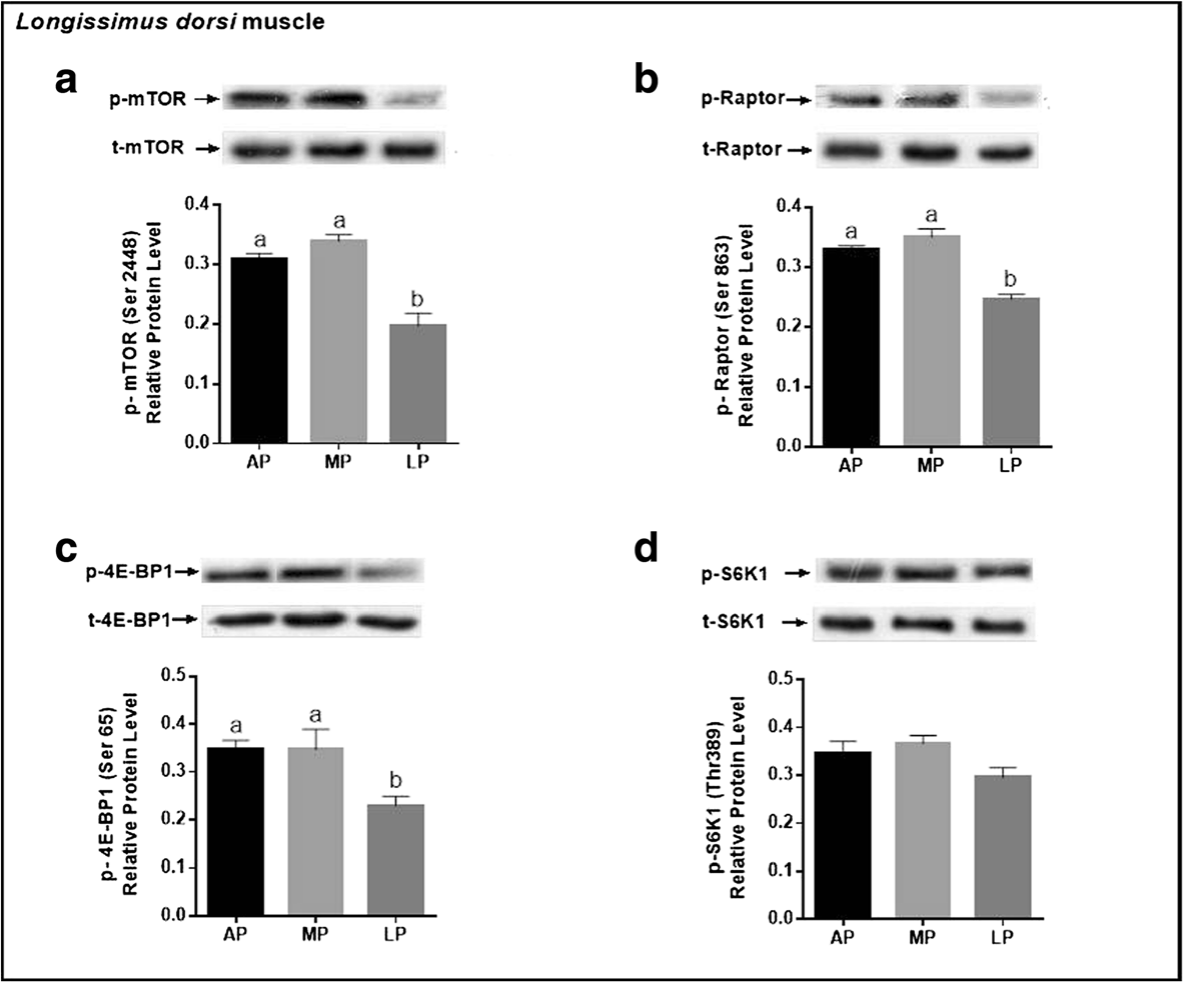
Phosphorylated protein expression levels of key molecules for mTORC1 signaling pathway (mTOR, Raptor, 4E-BP1, S6K1) in the *longissimus dorsi* muscle of growing-finishing pigs fed the restricted protein diets. Data were normalized to the value of corresponding total protein and expressed as means ± SE (*n* = 6). Bars with different letters (**a**, **b**) are significantly different at *P* < 0.05. AP = adequate protein; MP = moderately restricted protein; LP = low protein

**Fig. 4:**
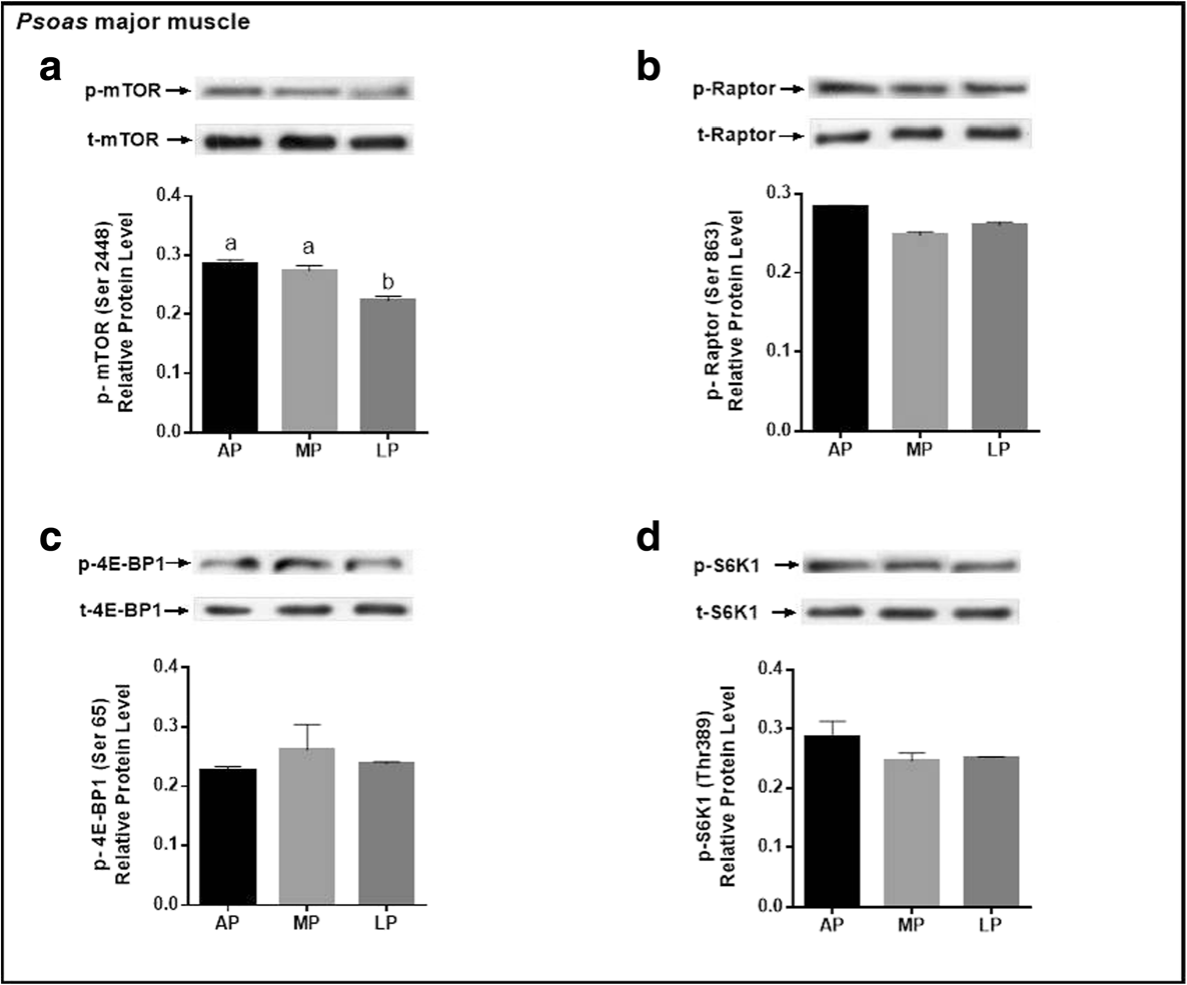
Phosphorylated protein expression levels of key molecules for mTORC1 signaling pathway (mTOR, Raptor, 4E-BP1, S6K1) in the *psoas major* muscle of growing-finishing pigs fed the restricted protein diets. Data were normalized to the value of corresponding total protein and expressed as means ± SE (*n* = 6). Bars with different letters (**a**, **b**) are significantly different at *P* < 0.05. AP = adequate protein; MP = moderately restricted protein; LP = low protein

**Fig. 5:**
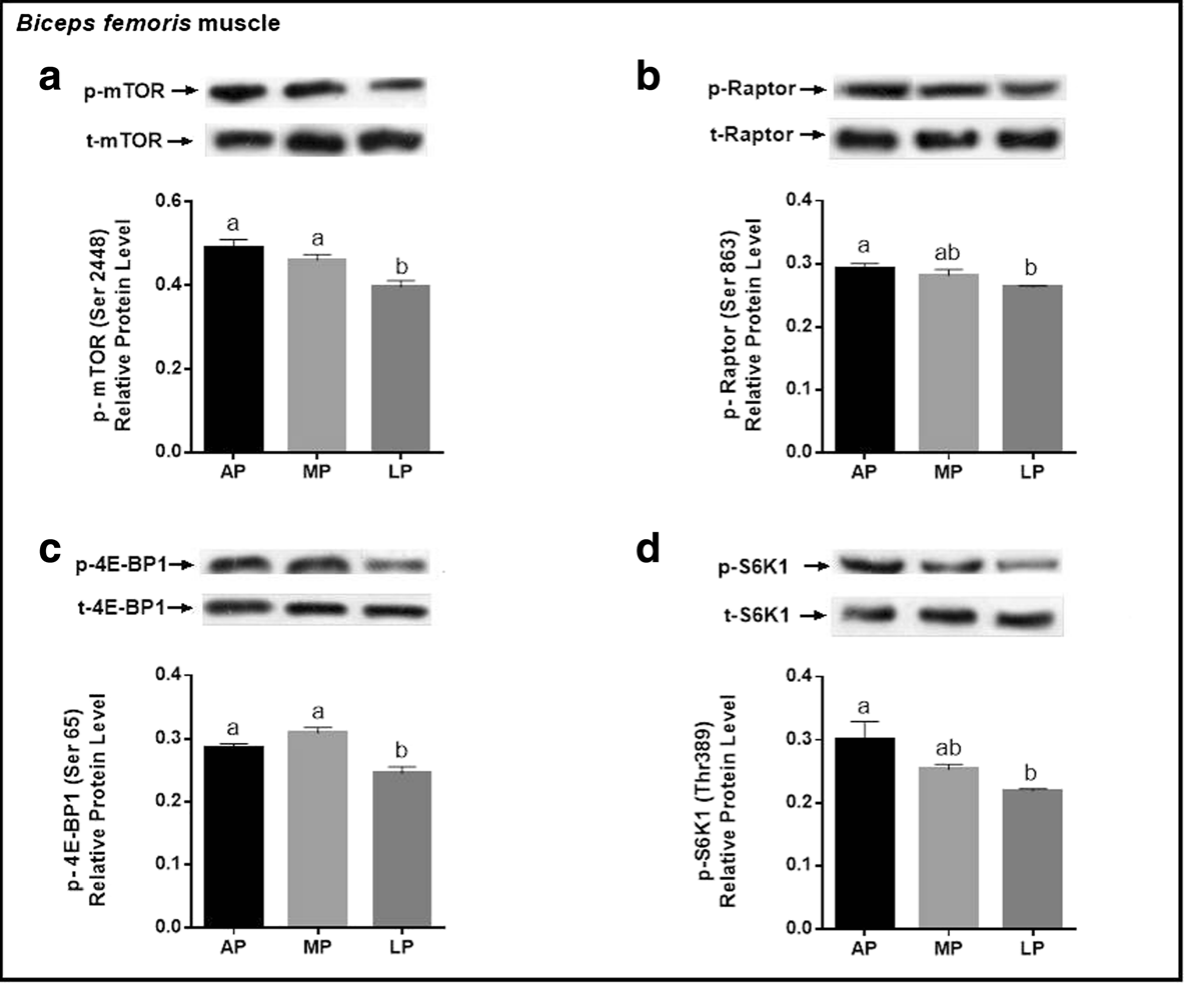
Phosphorylated protein expression levels of key molecules for mTORC1 signaling pathway (mTOR, Raptor, 4E-BP1, S6K1) in the *biceps femoris* muscle of growing-finishing pigs fed the restricted protein diets. Data were normalized to the value of corresponding total protein and expressed as means ± SE (*n* = 6). Bars with different letters (**a**, **b**) are significantly different at *P* < 0.05. AP = adequate protein; MP = moderately restricted protein; LP = low protein

As shown in Fig. 3, in LDM, pigs fed the LP diet had the lowest (*P* < 0.05) protein level of p-mTOR, p-Raptor and p-4E-BP1 compared with those fed the AP or MP diet, but there was no difference in the protein level of p-S6K1 among the treatments. Moreover, the protein level of p-mTOR was markedly enhanced (*P* < 0.05) in PMM of AP or MP-fed pigs, but the value of p-Raptor, p-4E-BP1 and p-S6K1 in PMM were not altered by dietary treatments (Fig. 4). In BFM, the protein level of p-mTOR and p-4E-BP1 were decreased (*P* < 0.05) in LP-fed pigs relative to the other two groups; the value of p-Raptor and p-S6K1 were reduced (*P* < 0.05) in pigs fed the LP diet relative to those fed the AP diet, but there was no difference between MP and AP groups (Fig. 5).

## Discussion

The present study showed that feeding the LP diet (10 % CP) to the growing-finishing pigs significantly retarded the growth performance even though the diet was supplemented with the limited amino acids. However, feeding the MP diet (13 % CP) restored the growth performance of pigs compared with those fed the LP diet. Furthermore, the MP diet improved the feed conversion efficiency of pigs to a level similar to that of AP diet (16 % CP), which is consistent with the previous studies that the growth response was unaffected in pigs fed the slightly low-protein diet [32–37]. In addition, a trend to significance was observed in the LDM weight of the MP-fed pigs relative to the other two groups. Overall, the results of the current study suggests that moderately restricted CP level in the diet would not negatively influence growth performance of the growing-finishing pigs, but an excessively reduced CP level in the diet could impair growth and development of the pigs.

The skeletal muscle, including LDM, PMM and BFM, is a highly heterogeneous tissue, mainly composed of myofiber types: oxidative (I and IIa), glycolytic (IIb) and intermediate (IIx) [38–40]. The tissues of LDM, PMM, BFM mainly contain the fiber type of IIb, whereas PMM contains higher oxidative type (I and IIa) than LDM or BFM, and for BFM, the proportion of type I fiber is higher than LDM, but for other fiber types the proportion is almost intermediate (Additional file 1: Table S1). The pigs fed the MP diet up-regulated mRNA expression of the oxidative isoform-MyHC IIa in the three types of skeletal muscle compared with those fed the LP- and/or AP diet, and the value of MyHC I in pigs fed the MP diet was higher than that of the LP-fed pigs in BFM only, whereas the mRNA levels of type IIb and IIx were unaffected by dietary CP level. Similar results have been reported previously that the proportion of oxidative isoforms was higher, while MyHC IIb was lower in the muscle of pigs fed the low CP diet than those fed the high CP diet [41–43]. Oxidative isoforms (I and IIa) fibers have a better aerobic ability [44] and a higher capacity to synthesize protein [45]. Furthermore, several studies have found that fiber type composition produces important impacts on meat quality, for example, the increasing proportion of MyHC I fiber could be expected to improve tenderness of meat [46]. Based on these, our findings indicate that feeding the MP diet to growing-finishing pigs might have a potential improvement of myofiber phenotype which is beneficial to meat quality.

Myofiber isoforms composition influences the biochemical and metabolic properties along with energy metabolism in the skeletal muscle of livestock animals and humans [47–49]. Thus we evaluated the energy status of LDM, PMM and BFM via determining the content of ATP, ADP, AMP and EC value. Interestingly, the values of those parameters were altered differently in various skeletal muscle locations of pigs fed the restricted protein diets. In specific, the highest ATP content and EC value were observed in LDM of the MP-fed pigs, PMM of the AP-fed pigs and BFM of the LP-fed pigs, respectively. Generally, low energy status induces AMP-activated protein kinase signaling pathway, the subsequent increase in the rate of myofiber degradation limits the fiber size and inhibits the mTORC1 pathway to facilitate the restoration of cellular energy status [50]. It means that in order to maintain energy homeostasis, intracellular lower energy status could inhibit translational initiation and protein synthesis through down-regulating mTOR pathway [51]. In another way, the energy status limitation has also linked to protein degradation *via* increasing the expression level of E3 ligases (MuRF1 and MAFbx) [52]. Unexpectedly, in this study, restricted protein diet did not alter the mRNA abundance of *MuRF1* and *MAFbx* in all three locations of the skeletal muscle.

Multiple data have indicated that the myogenic regulatory factors (MRFs) -MyoD and MyoG- relevant to muscle development and plasticity. The MyoD acts early in myogenesis to determine myogenic fate, while MyoG functions later in the differentiation of myoblasts into myotubes [3]. Besides, MyoD is a potent activator of fast transcription in skeletal muscle, and it also directly acts on type II MyHC promoters and other fibre-type-specific promoters [32]. Our results showed that the MP diet induced higher mRNA levels of *MyoD* and *MyoG* in BFM but not in LDM and PMM. It is likely that the increase of MyHC IIa responded to the MP diet was concomitant with the up-regulation of MRFs in the pigs. It is widely accepted that the expression level of myokine IL-6 is relatively high in the oxidative muscle fiber [53]. Myokine IL-15, as a novel anabolic factor for skeletal muscle, is able to stimulate the accumulation of contractile proteins in the differentiated myocytes or muscle fibers [54], suggesting its critical role in muscle fiber development. In our experiment, the mRNA levels of *IL-15* in the different locations of muscle tissue (LDM, PMM and BFM) were all markedly enhanced in pigs fed the MP diet compared to those fed the LP- and/or AP diet, indicating MP-fed pigs had a high capacity of protein deposition in the muscle tissue.

The activation of mTOR has been considered as nutrient and energy sensor of the cell and plays a prominent role in the regulation of protein synthesis [55–57]. The Raptor is a core component of mTORC1 primarily mediating cellular growth in response to anabolic stimuli. In the present study, we found that protein levels of p-mTOR were all up-regulated in the LDM, PMM and BFM of pigs fed the MP or AP diet compared with those fed the LP diet. Tendencies of the protein levels of p-Raptor and p-4E-BP1 were the same as that of p-mTOR in LDM, but in PMM there were no difference in the expression levels of the selected protein among treatments except for p-mTOR; in BFM, the protein level of p-4E-BP1 exerted the same tendency as p-mTOR, however, the values of p-Raptor and p-S6K1 were higher in AP-fed pigs than LP-fed pigs and intermediate in the MP-fed pigs. Collectively, the degree of mTOR pathway activation is not similar in skeletal muscle of different fiber type responding to the dietary protein level in the growing-finishing pigs. The current study is in agreement with a previous study which reported different sensitivity of various skeletal muscle fiber types in response to mTOR signaling molecules between plantaris and soleus [58]. Based on the current results, we speculated that the MP diet-induced muscle mass weight increase in LDM is at least partially mediated through the activation of the mTORC1 signaling pathway.

## Conclusion

In conclusion, our findings confirmed that the growing-finishing pigs chronically fed a moderately restricted protein diet (13 % CP) could catch up growth performance and LDM weight of the pigs offered the adequate protein level diet (16 % CP). It was partly attributed to the modulation of the fiber type characteristics and energy status, the up-regulated mRNA levels of myokines (*IL-6*, *IL-15*), MRFs (*MyoD*, *MyoG*), and the activation of mTORC1 pathway. These findings may provide new insight into the application of nutrition strategy in pig industry or even in human health.

## Abbreviations

ADFI, average daily feed intake; ADG, average daily gain; AP, adequate protein; BFM, *biceps femoris* muscle; CP, crude protein; eIF•4E-BP1, eukaryotic initiation factor 4E-binding protein1; IL-6, interleukin -6; LDM, *longissimus dorsi* muscle; LP, low protein; MAFbx, muscle atrophy F-box; MP, moderately restricted protein; MRFs, myogenic regulatory factors; mTORC1, mammalian target of rapamycin complex 1; MuRF1, muscle ring finger 1; MyHC, myosin heavy chain; PMM, *psoas* major muscle; S6K1, p70S6 kinase; UPP, ubiquitin proteasome pathway

## Additional file

Additional file 1: Table S1. Characteristics of skeletal muscle fiber (*longissimus dorsi* muscle, *psoas* major muscle and *biceps femoris* muscle) of male growing-finishing pigs^1^. (DOCX 15 kb)
