# Supplementary material for: Effects of dietary protein restriction on muscle fiber characteristics and mTORC1 pathway in the skeletal muscle of growing-finishing pigs
Source: J Anim Sci Biotechnol. 2016 Aug 22;7(1):47. doi: 10.1186/s40104-016-0106-8 (PMC4994323; doi:10.1186/s40104-016-0106-8)
Supplement: Additional file 1: Table S1. — Characteristics of skeletal muscle fiber (longissimus dorsi muscle, psoas major muscle and biceps femoris muscle) of male growing-finishing pigs1. (DOCX 15 kb) [file 40104_2016_106_MOESM1_ESM.docx]

**Supplementary Table 1. Characteristics of skeletal muscle fiber (*longissimus dorsi* muscle, *psoas* major muscle and *biceps femoris* muscle) of male growing-finishing pigs^1^**

| **Items** | **Skeletal muscle^2^** | | |
| --- | --- | --- | --- |
|  | **LDM** | **PMM** | **BFM** |
| **Fiber type proportions** | | | |
| I | 0.12 | 0.25 | 0.24 |
| IIa | 0.07 | 0.17 | 0.08 |
| IIx | 0.005 | 0.019 | 0.003 |
| IIb | 0.81 | 0.56 | 0.67 |
| **Cross-sectional areas (μm^2^)** | | | |
| I | 2775 | 2413 | 2765 |
| IIa | 2164 | 2212 | 3103 |
| IIb | 4467 | 3683 | 5315 |
| Mean | 4082 | 3077 | 4447 |
| **Capillarization** | | | |
| caplillaries per fiber | 1.3 | 1.3 | 1.7 |
| caplillaries per mm^2^ | 331 | 420 | 427 |

^1^The data are reported from Petersen JS, Henckel P, Oksbjerg N, and Sørensen M T (1998). Characteristics of muscle fiber type were determined using ATPase staining and α-amylase-PAS staining.

^2^LDM = *longissimus dorsi* muscle; PMM = *psoas* major muscle; BFM = *biceps femoris* muscle.
